# Proactive psychological programs designed to mitigate posttraumatic stress injuries among at-risk workers: a systematic review and meta-analysis

**DOI:** 10.1186/s13643-021-01677-7

**Published:** 2021-04-28

**Authors:** Paula M. Di Nota, Anees Bahji, Dianne Groll, R. Nicholas Carleton, Gregory S. Anderson

**Affiliations:** 1 Department of Psychology, University of Toronto, Toronto, Canada; 2 Department of Psychiatry, University of Calgary, Calgary, Canada; 3 Department of Psychiatry, Queen’s University, Kingston, Canada; 4 Department of Psychology, University of Regina, Regina, Canada; 5 Faculty of Science, Thompson Rivers University, TRU Way, Kamloops, BC V2C 0C8 Canada

**Keywords:** Posttraumatic stress injuries, Organizational stress, Mental health training, Occupational health, Resilience, Emergency personnel, Essential workers, Public safety, Healthcare, Meta-analysis

## Abstract

**Background:**

Public safety personnel and frontline healthcare professionals are at increased risk of exposure to potentially psychologically traumatic events (PPTE) and developing posttraumatic stress injuries (PTSI, e.g., depression, anxiety) by the nature of their work. PTSI are also linked to increased absenteeism, suicidality, and performance decrements, which compromise occupational and public health and safety in trauma-exposed workers. Evidence is lacking regarding the effectiveness of “prevention” programs designed to mitigate PTSI proactively. The purpose of this review is to measure the effectiveness of proactive PTSI mitigation programs among occupational groups exposed to PPTE on measures of PTSI symptoms, absenteeism, and psychological wellness.

**Methods:**

Five electronic databases were searched per PRISMA guidelines for English or French peer-reviewed studies from 2008 to 2019 evaluating PTSI and psychological wellness in adults exposed to occupational PPTE. The risk of bias was assessed using the Newcastle-Ottawa Scale.

**Results:**

We identified 42 studies evaluating 3182 public safety and frontline healthcare professionals, PPTE-exposed educational staff, and miners. Significant overlap was found across program themes that included mindfulness, psychoeducation, resilience promotion, and stress management strategies. Post-program effect sizes were small (*SMD* < 0.5) to moderate (*SMD* < 0.8) for reductions in PTSI symptoms and for promoting measures of well-being as indicated by a meta-analysis on 36 studies. There was no evidence for significant reductions in substance use, absenteeism, or biomarkers of distress except for heart rate. Subgroup analyses indicated that multimodal programs effectively improved general psychological health, while resilience programs improved measures of depression, burnout, coping, and resilience. Effect sizes for resilience, depression, and general psychological health improvements were greatest immediately or 1-month post-training, while improvements in PTSD symptoms and coping were larger at longer follow-up. Studies were of moderate quality and risk of bias.

**Conclusions:**

The current results showcase modest evidence for time-limited reductions in PTSI following participation in holistic programs that promote resilience, stress, and emotion regulation among at-risk workers. Implications for organizational implementation of proactive PTSI mitigation programs and areas of future research are discussed.

**Systematic review registration:**

PROSPERO (CRD42019133534)

**Supplementary Information:**

The online version contains supplementary material available at 10.1186/s13643-021-01677-7.

## Background

Public safety personnel (PSP) serve to maintain public safety and well-being. Occupations included within the definition of PSP include, but are not limited to, border services officers, public safety communications officials (e.g., dispatch or 911 operators), correctional workers, firefighters (career and volunteer), paramedics, and police [1]. PSP and frontline healthcare personnel (FHP, e.g., nurses, physicians, social workers, counselors, and staff in emergency, trauma, surgical, psychiatric, geriatric, and/or intensive care units) are frequently and repeatedly exposed to potentially psychologically traumatic events (PPTEs) [1–3]. Consequently, PSP and FHP appear to be at increased risk for posttraumatic stress injuries (PTSIs) [4–7], which appears to be further exacerbated during the global COVID-19 pandemic [8–11].

PTSI typically include symptoms of major depressive disorder, panic disorder, generalized anxiety disorder, posttraumatic stress disorder, suicidal ideation and attempts, and substance abuse [1, 4, 12]. In a recent pan-Canadian survey of PSP, 44.5% of respondents screened positive for at least one occupationally mediated PTSI [4, 13]. Furthermore, PSP appear up to four times more likely than the general population to report suicidal behaviors (i.e., ideation, planning, attempts, deaths) [14, 15]. Recent evidence for PTSI prevalence among FHP is lacking; however, the Canadian Federation of Nurses Unions [16] reported that 61% of nurses had experienced abuse, harassment, or assault in the workplace. FHP also report high levels of occupationally mediated compassion fatigue and burnout [17]. The concept of burnout was first proposed in the early 1970s by psychologist Christina Maslach, who explored a phenomenon among care providers involving emotional exhaustion, depersonalization, and diminished personal achievement [18]. Over time, this tripartite construct has become known as burnout [19]. Burnout is currently not a diagnosable mental health disorder but has been formally included as a problematic syndrome in ICD-11 [20].

Evaluations of PPTEs among PSP and FHP have focused mainly on first responders and frontline workers; however, recent evidence indicates that the civilians who work alongside them (e.g., administrative staff, public service employees, victim services) are also frequently exposed to PPTE and report comparable levels of PTSI and suicidal behaviors [21]. The COVID-19 pandemic has also highlighted several less conventional “essential” occupational sectors at increased risk of occupational PPTEs, including public-facing personnel such as transportation workers, grocery clerks, and restaurant workers [22]. Workers in extractive sectors including miners and drillers also regularly face life-threatening operational conditions, increasing the risk for occupationally mediated PPTE [23]. While any study of the effectiveness of a proactive psychological program delivered in an occupational context will qualify for inclusion in the current work, we will focus on PSP and FHP (broadly defined) as the extant literature supports that these occupational groups are most frequently exposed to work-related PPTE.

PTSI symptoms may also negatively impact occupational performance quality, increase absenteeism, increase sleep difficulties, negatively impact interpersonal relationships, increase burnout, and increase early mortality [4, 12, 24, 25]. The economic burden of PTSI among Canadian PSP and FHP is unknown [26]; nevertheless, annual productivity losses from mental disorders experienced by Canadians are estimated to cost between $16.6 [27] and $21 billion [28]. In the USA, health care costs for treating a firefighter, paramedic, or police officer with PTSD are almost five times higher than one without PTSD (~ $10,000/year versus ~ $2000) [26]. The significant costs have prompted several stakeholder organizations and occupational health policymakers to seek proactive approaches, such as implementing psychological and mental health training programs to mitigate the impact of PPTE on workers [29]. Accordingly, psychological interventions that promote well-being have been shown to reduce absenteeism [30, 31]. Proactive measures to support mental health may be particularly relevant for PSP given evidence that stigma is substantially inhibiting care-seeking for mental health challenges [13].

Proactive psychological programs have occasionally been integrated into basic training as part of efforts to increase individual resilience before PPTE exposures, as demonstrated among paramedic [32, 33] and nursing students [34], as well as federal and special forces police in Canada and abroad [35–37]. Proactive psychological programs have been increasingly offered to experienced workers who have already been exposed to PPTE but are intended to “prevent” or mitigate the development of PTSI rather than treat them. The current systematic review and meta-analysis focuses on a broad variety of proactive psychological program types in order to investigate the degree to which various occupationally mediated PTSIs are impacted by different programming approaches. We are reticent to label “prevention” programs because of rampant misuses of the term in the existing literature and mental health programming. A program can only be deemed preventative with highly rigorous pre- and post-training PTSI clinical screenings among persons who do not already have a PTSI or mental disorder, which would confound the results. The extant literature indicates that any post-training gains (i.e., effect sizes) are small and very time-limited [13, 38]; also, the gains are expected to deteriorate like other learned skills [33, 39, 40], meaning refresher programs are likely critical for maintaining gains. Despite important efforts at summarizing the existing pre- and post-exposure programming options for first responders frequently exposed to PPTE [41–43], there are currently significant research gaps regarding the effectiveness of proactive programs designed to mitigate PTSI, especially among FHP.

### Objectives

The current study was designed as a systematic literature review to identify published research on proactive PTSI mitigation programs tailored for PSP, FHP, and other workers exposed to PPTE. The effectiveness of such programs for improving outcomes related to PTSI and psychological health will be evaluated with a quantitative meta-analysis. Comparators will include controls in the waitlist, nil treatment, training as usual, or alternative programming groups, and baseline scores for within-subject studies. Results are presented to summarize the various training approaches, durations, and outcomes evaluated in empirical studies of program effectiveness. The current results can assist industrial, organizational, and occupational stakeholders in implementing evidence-based programming for mitigating PTSI among at-risk workers.

## Methods

### Protocol and registration

**Fig. 1:**
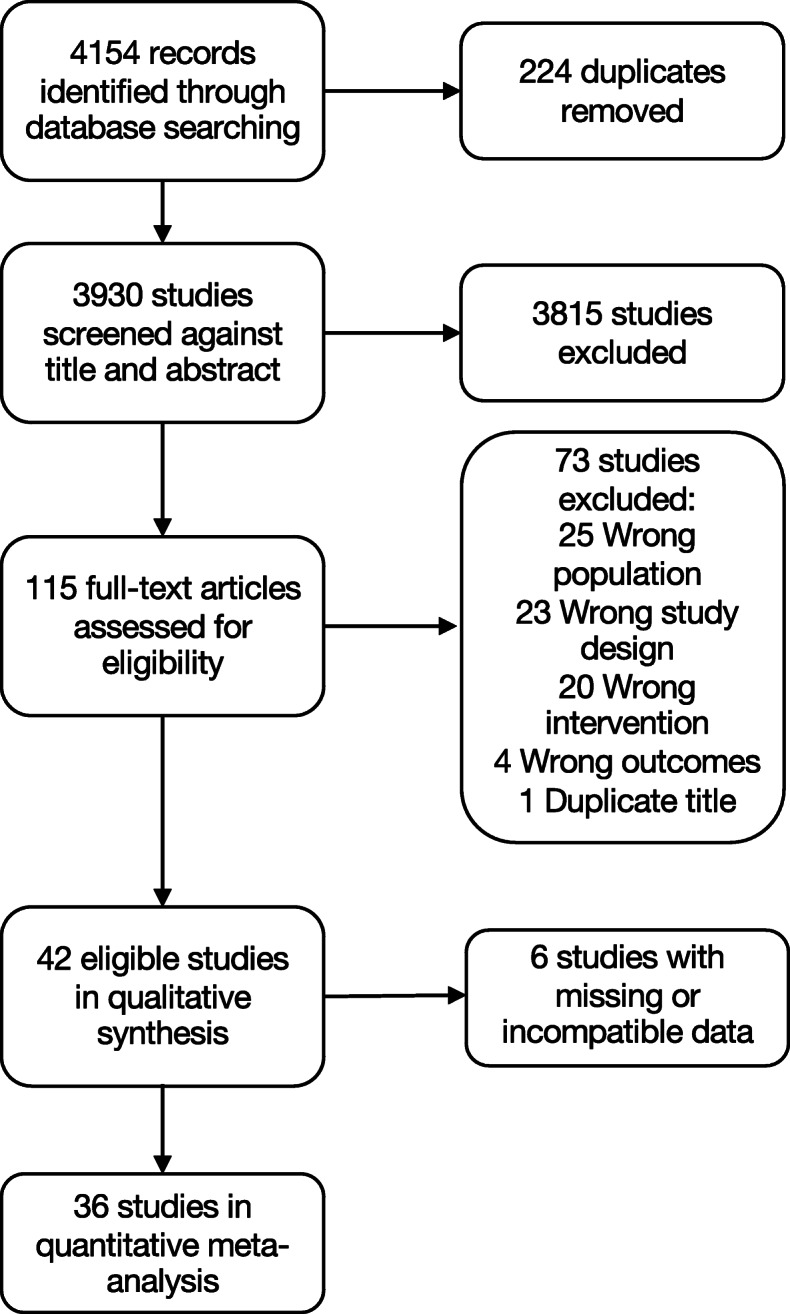
PRISMA flow diagram

The current study was pre-registered with PROSPERO (CRD42019133534) [44]. Systematic literature review procedures followed PRISMA guidelines [45], as illustrated in Fig. 1 and in the PRISMA checklist in **Additional File** 1.

### Eligibility criteria

The current review was restricted to peer-reviewed English- or French-language studies assessing the impact of any mental health program designed to mitigate the impact of PPTE among adult (aged 18 and older) workers and published since January 1, 2008. To maximize yield, we extended eligibility to any PPTE-exposed group of workers, including counselors, correctional workers, dispatchers, emergency workers, firefighters, nurses, paramedics, police, rail transit operators, and social workers. Eligible study designs included randomized control trials (RCT) and quasi-experimental studies (e.g., pre-post studies). Studies involving participants with one or more identifiable mental disorders (e.g., clinician diagnosis or a positive screen on a validated psychological instrument), non-PPTE occupational stressors (e.g., work-related demands, organizational stress), or non-experimental designs (e.g., protocols, theses, qualitative studies) were excluded.

### Information sources

A population-intervention-comparison-outcome (PICO) framework was used to define study variables of interest and keywords entered into our systematic literature searches, which are provided in Table 1. We searched EMBASE, MEDLINE, PsycINFO, PubMed, and Web of Science between 2008 and December 9, 2019. The database-facilitated searches were supplemented for additional studies with hand-searches of the reference lists from included studies, as well as previous review articles and reports. Following the searches, all citations were imported into Covidence—a web-based systematic review manager [46]. There were two independent reviewers who screened articles against the eligibility criteria: first by title/abstract and then in full. Initial screening was verified by having multiple reviewers screen 200 papers resulting in 99% agreement. All discrepancies were resolved by consensus between the two reviewers.

**Table 1:** PICO literature search strategy

Domain	Target	Search terms
Population	Public safety personnel	Firefighters Police officers Law enforcement Dispatch Communication officers Paramedic Emergency medical technician Emergency medical service First responders Correctional officers Emergency workers Emergency response team Emergency room personnel Nurses Transit operators Transit workers Social workers Counselors
Intervention	Prevention training programs	Prevention Resilience Coping (skills) Family coping Stress reduction Skill building Wellness capacity Capacity building Psychoeducation Mental health awareness (training) Stigma reduction
Comparison	Control group	Waitlist control Randomized control trial
Outcome	Posttraumatic stress injuries	Operational Stress Injury PTSD PTSI Occupational stress Trauma Trauma exposure (Major) depression Anxiety Substance use disorder Chronic pain Insomnia Stress

### Data extraction

There were two reviewers who extracted data independently from published full-text reports of eligible articles. Per the PICO framework (Table 1), population variables included sample size, age, sex, and years of employment. Intervention variables included the duration of the training program, as well as program themes and approaches reported by study authors. Comparison variables included the type and nature of the comparator group. Outcome variables included absenteeism, scores on validated psychological instruments (i.e., General Health Questionnaire [GHQ], Symptoms Checklist 90 [SCL-90], Depression Anxiety Stress Scale-21 [DASS-21], and physiological markers of stress (e.g., heart rate, blood pressure, salivary and plasma cortisol). Absenteeism did not include individuals already on medical leave at the time of the study. Absenteeism was measured for individuals participating in the PTSI mitigation program (versus annual reports of overall sickness absence). Missing data or outcomes reported in incompatible form for the meta-analysis (e.g., ranks, medians, regression results) were requested from corresponding authors. To maximize power for the meta-analysis, program types and outcome variables were categorized, and operational definitions are provided in Tables 2 and 3, respectively.

**Table 2:** Proactive PTSI mitigation program categories and specific interventions included in the meta-analysis

Intervention category	Specific programs included
Emotion Regulation	1. Emotion Regulation Training
Mindfulness-based	1. Yoga 2. Mindfulness-Based Resilience Training 3. Mindfulness-Based Stress Reduction
Resilience Promotion	1. Resiliency Training Program 2. Online Resiliency Training 3. Imagery and Skills Training 4. Complementary Psychological Training 5. International Performance Resilience and Efficiency Program (iPREP)
Multimodal	1. Relation, mindfulness, CISD 2. ERASE-Stress intervention 3. Work-related gratitude diary 4. Eclectic group counseling 5. Stress Management (multimodal) 6. Integrated Health Program
Stress Management	1. Acceptance and Commitment Therapy
Web-based psychoeducation	1. Online Workplace Mental Health Intervention 2. Web-based stress management program 3. Road to Mental Readiness 4. Stress Management Mobile App

**Table 3:** Outcome categories and specific measures included in the meta-analysis

Outcome category	Specific measures included	Direction
Absenteeism	1. Number of days on sick leave in previous 2 months 2. Number of weeks on full-time sick leave the preceding year 3. Number of days on sick leave	Lower is better
Alcohol	1. Patient Reported Outcomes Measurement Information System (PROMIS) Alcohol Use Subscale 2. Alcohol use disorders identification test (AUDIT) 3. Drank in the past 12 Months 4. Number of days having 5 or more drinks on one occasion in past 30 days 5. Number of drinks per drinking day in past 30 days 6. Using Alcohol to Relieve Stress	Lower is better
Anger	1. Personal and Organizational Quality Assessment: anger and resentment subscale	Lower is better
Antithrombin	2. Serum antithrombin	Lower is better
Anxiety	1. Patient Reported Outcomes Measurement Information System (PROMIS) Anxiety Subscale 2. Depression Anxiety Stress 21 Scale (Anxiety subscale) 3. State-Trait Anxiety Inventory (STAI) 4. Profile of Mood States Tension-Anxiety Subscale 5. Brief Symptom Inventory: Anxiety Subscale 6. Personal and Organizational Quality Assessment-Anxiety Subscale 7. General Health Questionnaire-Anxiety Subscale 8. Hospital Anxiety and Depression Scale-Anxiety Subscale 9. Symptoms Checklist (SCL-90)-Phobic Anxiety Subscale 10. Adult Manifest Anxiety Scale 11. DASS-21: Anxiety	Lower is better
Blood pressure	1. Diastolic blood pressure 2. Systolic blood pressure	Lower is better
Burnout	1. Maslach Burnout Inventory Depersonalization Domain 2. Maslach Burnout Inventory Emotional Exhaustion Domain 3. Maslach Burnout Inventory Personal Accomplishment Domain 4. Oldenburg Burnout Inventory 5. Professional QoL: Burnout 6. Professional Quality of Life Scale 7. Maslach Burnout Inventory Overall	Lower is better
Coping	1. Emotion-Regulation Skills Questionnaire 2. Brief-Coping Orientation to Problems Experienced (Brief-COPE) 3. Operationalized 3-item coping skills measure 4. Recovery Experiences Questionnaire-Global Score	Higher is better
Cortisol	1. Serum cortisol 2. Salivary cortisol	Lower is better
Depression	1. PANAS-Negative Affect Subscale 2. PANAS-Positive Affect Subscale* (higher is better) 3. DASS-21 Scale (Depression subscale) 4. Brief Symptom Inventory: Depression Subscale 5. Hospital Anxiety and Depression Scale-Depression subscale 6. Centre for Epidemiological Studies Depression Scale (CES-D) 7. Beck Depression Inventory II 8. Personal and Organizational Quality Assessment: anxiety and depression subscale 9. Profile of Mood States Negative Mood Composite	Lower is better
DHEA	1. Salivary dehydroepiandrosterone (DHEA) 2. Serum dehydroepiandrosterone-sulfate (DHEA-s)	Lower is better
Drug Use	1. Using prescription drugs as prescribed to relieve stress 2. Using prescription drugs not as prescribed to relieve stress	Lower is better
General Symptoms	1. General Health Questionnaire 2. Symptoms Checklist (SCL-90)-Overall	Lower is better
Heart rate	1. Average heart rate during scenarios 2. Average resting heart rate 3. High frequency (HF) heart rate variability (HRV) 4. Inter-beat Interval 5. Standard deviation of normal RR intervals 6. Maximum heart rate during scenarios 7. Heart rate recovery time	Lower is better
Prolactin	1. Serum prolactin	Lower is better
PTSD	1. Professional QoL: secondary traumatic stress 2. Posttraumatic Check List 3. Impact of events scale: intrusive subscale 4. Impact of events scale: avoidance subscale 5. Response to stressful experiences scale 6. Posttraumatic Diagnostic Scale 7. Posttraumatic Check List for DSM-5 (PCL-5)	Lower is better
Resilience	1. Freiburg Mindfulness Inventory 2. Connor-Davidson Resilience Scale 3. Acceptance and Action Questionnaire II 4. Brief Resilience Scale 5. Self-Compassion Scale 6. Resilience Scale-global score	Higher is better
Stress	1. Police Stress Questionnaire Organizational Subscale 2. Police Stress Questionnaire Operational Subscale 3. Perceived Stress Scale 4. Depression Anxiety Stress 21 Scale (Stress subscale) 5. Professional Quality of Life Scale 6. Job stress 7. Self-reported stress 8. Coping with stress: Full Scale 9. Symptoms of Distress: Full Scale	Lower is better
Suicidality	1. Concise Health Risk Tracking Scale (suicidal ideation)	Lower is better
Well-being	1. Health-Promoting Lifestyle Profile II 2. Patient-Reported Outcomes Measurement Information System (PROMIS) Global Scale 3. Professional QoL: Compassion satisfaction 4. Cognitive Fusion Questionnaire 5. Sources of support scale 6. Mental Health Continuum Short Form (MHC-SF): Overall 7. Performance-based self-esteem scale 8. Nurses Job Satisfaction	Higher is better

### Quality assessment

Study quality was appraised using the Newcastle-Ottawa Scale [47], which evaluates nine items across three domains: outcome, selection, and comparability. Each item received a rating of high, low, or unclear risk of bias; each instance of a low risk of bias counted as one point, for a total possible score of nine. Overall study quality was operationalized using the total score: scores of 9 as “high quality,” scores of 7 or 8 as “moderate to high quality,” scores of 5 or 6 as “moderate to low quality,” and scores of 4 and below as “low quality.”

### Synthesis of results

Eligible studies for the quantitative meta-analyses needed to report means and standard error or standard deviation values for study outcomes of interest (see Table 3). A random-effects model was applied to pool effect sizes across studies using standardized mean differences (*SMD*) and their corresponding 95% confidence intervals (CI). Cohen’s criteria [48, 49] were used to interpret an *SMD* of 0.2 as “small,” 0.5 as “medium,” and 0.8 or greater at “large.” *SMD*s were measured at all available post-training and follow-up timepoints.

### Assessment of heterogeneity and additional analyses

Heterogeneity was quantified using the *I*^2^ statistic [50] and forest plots to graphically display summary effect sizes across studies [51]. Outcomes with at least ten studies were explored for sources using the following pre-specified subgroup analyses: occupation (e.g., firefighters, police officers), intervention (e.g., mindfulness-based, multimodal), and timeline (e.g., post-training, 1-month follow-up, 18-month follow-up). Sensitivity analyses included comparisons of random-effects and fixed-effects model effect sizes, as well as with the leave-out-one technique. For outcomes with at least ten studies, publication bias was assessed using funnel plots, the trim and fill method [52], and Egger’s test of funnel plot asymmetry [53, 54].

## Results

### Systematic literature review

The systematic review identified a total of 4154 studies. Among the identified studies, there were 224 removed as duplicates, leaving 3930 studies for the title and abstract screening. There were 3815 records removed, leaving 115 studies for full-text review. There were 73 studies excluded at the full-text stage: 25 had a wrong population (i.e., not PSP, FHP, or a PPTE-exposed occupational group), 23 had a wrong study design (i.e., not a pre-post evaluation of outcomes such as qualitative studies or protocols, or non-peer-reviewed dissertations, books, or reports), 20 had a wrong intervention (e.g., post-PPTE service, treatment, or therapeutic intervention), 4 had wrong outcomes (e.g., program acceptability or outcomes unrelated to mental health or wellness), and 1 was a duplicate title. The systematic review process resulted in 42 eligible studies that evaluated the effectiveness of a proactive PTSI mitigation program in workers exposed to PPTEs. Key study characteristics are described below and are summarized in Table 4, including participant summaries, study designs, PTSI mitigation program themes, primary outcomes, and results. A subsequent six studies were excluded from the meta-analysis for failing to report the means and/or standard deviations for their primary outcome measures [72, 86, 89], or reporting the means and/or standard deviations in formats that were incompatible for a quantitative meta-analysis; for example, reporting regression results [71], medians [81], or ranks [77]. All authors were contacted with data requests, but data were not yet provided at the time of submitting the current review. Ultimately, 36 studies were included in a quantitative meta-analysis (Fig. 1).

**Table 4:** Summary characteristics of eligible studies (*n* = 42). Studies not included in meta-analyses (*n* = 6) are marked with an asterisk (*)

Study (quality)	Sample size	Population (country)	Design	Program description	Program duration	Evaluation	Outcomes	Results
Alexander et al., 2015 [55] (Moderate-Low)	40	Nurses (USA)	RCT	Mindfulness-based stress management (Yoga) vs. Nil training	1 session (time not provided) × 8 weeks	Pre-training, post-training	HPLP-II; FMI; MBI EE, DP, PA subscales	Significant post-training improvements in self-care (HPLP-II), EE and DP
Andersen et al., 2015 [35] (Moderate-Low)	18	Special forces (SWAT) police officers (Finland)	Prospective cohort study	Psycho-educational and physiological resilience promotion with HRV-BF (iPREP)	5 days × 60 min sessions + 15 min daily breathing practice	Pre-training, post-training	HR_max_; HR_avg_; respiratory achievement and coherence while listening to critical incident scenarios	Significant reductions in HR_avg_ and improvement in respiratory achievement scores on Day 5 vs Day 1 of training, indicative of improved autonomic regulation under stress
Andersen & Gustafsberg, 2016 [36] (Moderate-High)	12	Special forces (SWAT) police officers (Finland)	RCT	Psycho-educational and physiological resilience promotion with HRV-BF (iPREP) vs. TAU	5 days total: 2 pre-post evaluation days, 3 training days	Pre-training, post-training	HR_max_; HR_Rec_ to HR_Base_; BP; self-reported stress	At post-training evaluations, the iPREP group had significantly lower HR_Max_ (scenario 1 only)
Andersen et al., 2018 [40] (Moderate-low)	57	Police officers (Canada)	Prospective cohort study	Psycho-educational and physiological resilience promotion with HRV-BF (iPREP)	4 days total: 1.5 days of pre and post-training evaluation, 2.5 days of training	Pre-training, post-training, 6, 12, and 18 months	HR_Max_ and HR_Index_ during critical incident scenarios; HR_Rec_	Significant reductions in HR_Index_ at 12 months follow-up, but not maintained at 18 months follow-up; HR_Rec_ faster at 12 and 18 months follow-up relative to pre- and post-training and 6 months follow-up
Anderson, Vaughan & Mills, 2017 [33] (Moderate-Low)	138	Primary care paramedical students performing a duty practicum (Canada)	RCT	Web-based psychoeducational resilience promotion vs. TAU	6-8 hours	Pre-training, post-training	RS global score and subscales	Resilience training significantly improved all measures except meaningfulness subscale following in-field practicum. SD values obtained from authors.
Arble et al., 2017 [56] (Moderate-High)	22	Police officers (USA)	Prospective cohort study	Psychoeducational resilience promotion and coping skill building	5 × 90 min group sessions	Pre-training, 12 months	COPE subscales; Sources of Support Scale; PCL; HADS; AUDIT	Following their first year in the field, officers appeared to report improved use of positive reframing and humor, and significant reductions in anxiety
Arnetz et al., 2009 [57] (Low)	18	Police officers (Sweden)	RCT	Psycho-educational and physiological resilience promotion and coping skill building vs. TAU	2 h × 10 weeks	12 months	Serum antithrombin and cortisol; mean change in HR; self-reported stress; POMS vigor-activity subscale and negative mood composite	Following their first year in the field, trained officers appeared to report less negative mood, smaller changes in mean HR and self-reported stress, and greater changes in antithrombin following a simulated critical incident
Arnetz et al., 2013 [58] (Moderate-High)	75	Police officers (Sweden)	RCT	Psycho-educational and physiological resilience promotion vs. TAU	90 min × 10 weeks + homework 3× per week	Pre-training, 18 months	GHQ, serum cortisol; prolactin; DHEA	Statistically significant post-training improvement in GHQ only
Bademci et al., 2016 [59] (Moderate-Low)	42	Correctional officers (Turkey)	Prospective cohort study	Psychosocial support program	75-min sessions, 3 times a week × 11 weeks (41.25 hs total)	Pre-training, post-training	PANAS; MBI EE, DP, PA subscales; BDI; BAI	Significant post-training improvements on all measures
Berger et al., 2016 [60] (Moderate-High)	63	Educational staff affected by the 2011 Christchurch earthquake (New Zealand)	RCT	Multimodal psychoeducational resilience promotion (EZ) vs. Critical incident management (METI)	3 × 8 h sessions for both treatments	Pre-training, post-training, 8 months	PCL; ProQoL CF, burnout, CS subscales; CDRS	Resilience higher pre-training in EZ, significantly improved post-training for both groups. Greater improvements in PCL and ProQoL subscales for EZ compared with METI
Berking et al., 2010 [61] (Moderate-Low)	31	Police officers (Switzerland)	Crossover RCT	Psycho-educational and physiological emotion regulation, cognitive therapy, coping skill building (iTEC) vs. WLC	12 × 45 min sessions delivered on 3 days over 4 weeks + at least 3 brief and one longer daily homework	Pre-training, post-training	ERSQ; PANAS	Statistically significant post-training increase in ERSQ scores and near-significant increase in positive affect scores
Bolier et al., 2014 [62] (Moderate-Low)	366	Allied health professionals (nurses, surgery assistants, physiotherapists, radiotherapists) (The Netherlands)	Cluster RCT	Web-based psychoeducation vs. Nil training	4 to 8 weeks	Pre-training, 3 months, 6 months	MHC-SF global score + subscales; WHO-5 Well-being Scale; BSI depression and anxiety subscales	All measures except BSI depression improved post-training, 3 months and 6 months follow-up for both groups. Significant improvement in MHC-SF global and psychological well-being subscales for training group only. Very low uptake and compliance
Brinkborg et al., 2011 [63] (High)	106	Social workers (Sweden)	RCT	Psychoeducational stress management and cognitive therapy (ACT-SMI) in high stress (PSS ≥ 25) vs. ACT-SMI in low stress (PSS ≤ 24) vs. high-stress WLC vs. low-stress WLC	4 × 3 h biweekly group sessions + homework (physical exercise, mindfulness)	Pre-training, post-training	PSS; MBI global, EE, DP, PA subscales; GHQ; Pbse	Significant reductions in all measures except Pbse for all ACT-SMI participants compared with WLC. High stress groups: significant reductions in PSS, MBI. Low stress groups: significant reductions in PSS, MBI global + PA subscale only
Brondolo et al., 2017 [64] (Moderate-Low)	257	ME’s, investigators, autopsy technicians, clerics/administrators, laboratory workers, clergy, legal staff, and facilities managers (USA)	Prospective cohort study	Web-based psychoeducation	3 modules × 16 classes × 5-7 min each, mean completion = 10.69 classes (SD = 7.74, range 1-21)	3 months and immediately pre-training, 1 month post-training	BDI; PDS	Of the 76 participants who completed at least 8 classes, post-training BDI scores were significantly lower than baseline or pre-training values, no changes in PDS values. Unadjusted M and SD values not reported in the text but provided by authors.
Carleton et al., 2018 [38] (Moderate-Low)	133	Police officers (Canada)	Prospective cohort study	Psychoeducational resilience promotion, stress management, coping skill building (R2MR)	4-h group seminar	Pre-training, 6 months, 12 months	BRS; DASS subscales; PCL; AUDIT	No statistically significant changes in mental health or resilience post-training or at follow-up, but small significant post-training reductions in stigma
Cheng et al., 2015 [65] (Moderate-High)	102	Hospital workers (physicians, nurses, physiotherapists, and occupational therapists) (Hong Kong)	RCT	Emotion regulation	Gratitude journal (2 weekly entries × 4 weeks) vs. Hassle journal vs. Nil treatment	Pre-training, post-training, 3 months	CES-D, PSS	Significant post-training reductions for the gratitude group only, further improved (CES-D) or maintained (PSS) at follow-up. M and SD values extracted from reported regression analyses, author contacted for raw data
Chongruksa et al., 2012 [66] (Moderate-High)	42	Police officers (Thailand)	Cluster RCT	Multimodal psychoeducation and counseling vs. Mental health psychoeducation control	1.5–2 h/week × 12 weeks for both groups	Pre-training, mid-training, post-training, 1 month	BDI; GHQ global score + subscales; SCL-90 global score + subscales	Significant reductions in all measures mid- and post-training for the multimodal group only, and increased scores at 1-month follow-up
Christopher et al., 2016 [67] (Low)	43	Police officers (USA)	Prospective cohort study	Mindfulness-based resilience promotion	2 h × 8 weeks + 6 h final lesson + daily homework (20 h total)	Pre-training, mid-training, post-training	BRS; OLBI; PSQ; PSS; PROMIS Global Mental Health Subscale; cortisol AUC	Significant post-training improvements on all measures, and significant increase in cortisol AUC predicted by change in PROMIS mental health score
Christopher et al., 2018 [68] (Moderate-Low)	61	Police officers (USA)	RCT	Mindfulness-based resilience promotion vs. Nil training	2-h sessions × 8 weeks + 6-h session (20 h total)	Pre-training, post-training, 3 months	PROMIS subscales; Concise Health Risk Tracking Scale (suicidal ideation); PSQ; OLBI; CDRS; AUC cortisol	Significant post-training improvement in burnout and organizational stress only. Significant post-training reduction in cortisol AUC in males only. No differences at follow-up
Craigie et al., 2016 [69] (Moderate-High)	20	Nurses (Australia)	Prospective cohort study	Mindfulness-based resilience promotion	12 h total + daily mindfulness homework practice	Pre-training, post-training, 1 month	DASS subscales; ProQoL subscales; STAI; CDRS	Significant post-training reductions in DASS depression, ProQoL burnout, and STAI, only the latter two remained significant at follow-up. Significant improvements in DASS Stress and ProQoL compassion satisfaction from pre-training to follow-up
Daigle et al., 2018 [70] (Moderate-Low)	70	Nurses (Canada)	RCT	Mindfulness-based stress management vs. WLC	2.5 h × 8 weeks + full day retreat + recommended 45 min daily practice	Pre-training, post-training	POMS-TA	Significantly reduced POMS-TA post-training
Duarte et al., 2017 [71] (Low)*	48	Oncology nurses (Portugal)	Prospective cohort study	Mindfulness-based stress management	2-h group sessions × 6 weeks + 15 min daily homework	Post-training	DASS subscales; ProQoL BO, CF subscales, SLS	Significant post-training reductions in DASS Stress, ProQoL, and SLS. Direct effects of regression analyses reported only, author contacted for M and SD values
Duchemin et al., 2015 [72] (Moderate-Low)*	32	SICU personnel (USA)	RCT	Multimodal mindfulness-based intervention vs. WLC	9 × 1 h weekly sessions + recommended 20 min daily practice	Pre-training, post-training	PSS, DASS stress subscale, MBI EE, DP and PA subscales, ProQoL CS, BO STS subscales, self-report work stress	Significant post-training reduction in DASS stress and proportion of participants with high (> 26) EE scores. M and SDs for primary outcome measures not provided, authors contacted.
Flarity et al., 2013 [73] (Moderate-Low)	59	Nurses (USA)	Prospective cohort study	Psychoeducational resilience promotion	4-h group seminar	Pre-training, post-training	ProQoL CS, BO, STS subscales	Significant post-training improvements in all subscale scores and proportion in high/low cut-off ranges
Hersch et al., 2016 [74] (Moderate-Low)	104	Nurses (USA)	RCT	Web-based stress management (BREATHE) vs. WLC	7 online modules (average time = 43 min)	Pre-training, 3 months post-training	Nursing Stress Scale; Symptoms of Distress (emotional symptoms subscale); Coping with Stress Scale	Significant post-training improvement in Nursing Stress Scale only. Low rates of participation
Joyce et al., 2018 [75] (Low)	29	Firefighters (Australia)	Prospective cohort study	Web-based, mindfulness-based resilience promotion (RAW)	6 × 20–25 min sessions + optional practice	Pre-training, post-training	CDRS; CFQ; AAQ-II	Mean increase in resilience and reduction in cognitive fusion, psychological inflexibility, and avoidance, but not statistically significant
Joyce et al., 2019 [76] (Moderate-High)	143	Primary Fire and Rescue and Hazmat (Australia)	Cluster RCT	Web-based, mindfulness-based resilience promotion (RAW) vs. Healthy Living Program (control)	6 × 20–25 min sessions + optional practice vs. 6 × 20 min modules	Pre-training, 6 weeks and 6 months post-training	CDRS; BRS; FMI; AAQ-II; SCS; LOT-R; COPE active coping, emotional support, instrumental support subscales; LET	Significantly higher resilience and active coping in RAW participants at 6 months follow-up compared with control but coping not sustained at follow-up. Improved mindfulness sustained in full but not partial program completers. Authors contacted for CDRS and BRS SD values.
Larijani et al., 2018 [77] (Low)*	126	Red Crescent Healthcare Centers (Iran)	Cluster RCT	Resilience promotion vs. Nil training	No description	Pre-training, post-training	GHQ physical symptoms, anxiety, social dysfunction, and depression subscales	Post-training improvements in physical symptoms, anxiety, and social dysfunction in experimental group only. Ranked data not useable for meta-analyses, authors contacted for unadjusted M and SD values.
Lin et al., 2019 [78] (Moderate-Low)	90	Nurses (China)	RCT	Multimodal mindfulness-based cognitive therapy vs. WLC	8 × 2 h weekly group mindfulness sessions + recommended practice vs. nil	Pre-training, post-training, 3 months	PSS; PANAS; CDRS	Significant post-training improvements in perceived stress, positive affect, and negative affect maintained at follow up, improved resilience at follow-up compared with baseline
McCraty et al., 2009 [79] (Moderate-High)	75	Correctional officers (USA)	RCT	Psycho-educational and physiological stress management with HRV-BF (Power to Change Performance) vs. WLC	2 days + 3mons recommended practice at work	Pre-training, 3 months post-training	Salivary cortisol and DHEA; BP; HR_Rest_; HRV components (RMSSD, HF, LF, VLF, total power, LF/HF ratio); BSI subscales; POQA subscales	Significantly lower DHEA, BP, HR, anger and increased LF/HF HRV ratio post-training, no physiological changes, and increases in depressive symptoms in control group
McCraty & Atkinson, 2012 [80] (Moderate-Low)	59	Police officers (USA)	RCT	Psycho-educational and physiological resilience promotion stress management with HRV-BF (Coherence Advantage Program) vs. WLC	3 × 4 h sessions	Pre-training, post-training	POQA subscales; BP and IBI during critical incident scenarios (*n* = 23, 12 exp, 11 ctrl)	Depression declined by 13% among trained officers while it increased by 17% in the control group. Significantly greater decrease in IBI during the post-training scenario in experimental group only. Authors contacted for HR data (SDs) and POQA baseline scores
Mealer et al., 2014 [81] (Moderate-Low)*	27	ICU nurses (USA)	RCT	Multimodal psychoeducation, mindfulness-based practice, emotional regulation vs. Nil training	12 weeks total: 2-day workshop + 12 × 30 min weekly writing sessions + 15 min × 3/week mindfulness + 30–45min exercise × 3/week + 30–60 min counseling session	Pre-training, 1wk post-training	CDRS, PDS, HADS, MBI EE, DP, and PA subscales	Post-training reductions in depression symptoms in the experimental group. Both groups reported significant reductions in PTSD symptoms and improvements in resilience.
Molek-Winiarska & Żołnierczyk-Zreda, 2018 [82] (Moderate-High)	66	Miners (Poland)	RCT	Mindfulness-based stress management vs. Nil training	8-h sessions × 5 weeks (40 h total) + optional homework	Pre-training, 3 months post-training	GHQ global score and anxiety and depression subscales	Significant post-training reduction in anxiety and depression scores
Oliver & Meier, 2009 [83] (Moderate-High)	132	Small-town and rural police officers and sheriffs (USA)	Prospective cohort study	Stress management	8 h	Pre-training, post-training between 1-6mons, 7-12mons, or 13-18mons	Adult Manifest Anxiety Scale	No significant post-training reduction overall, but significant when analyzed according to post-test lag times (1-6mons, 7-12mons, 13-18mons)
Poulsen et al., 2015 [84] (Moderate-Low)	70	Radiation therapists and oncology nurses (Australia)	RCT	Stress management workshop vs. Written educational materials only	1 day	Pre-training, 6 weeks post-training	Recovery experiences questionnaire global score and subscales	Workshop group global scores increased post-training, and 3 of 4 subscales were higher than the control group
Ramey et al., 2016 [85] (Moderate-Low)	38	Police officers (USA)	Prospective cohort study	Psycho-educational and physiological emotion regulation with HRV-BF	2 × 2 h sessions held 2–3 weeks apart + 3mons practicing skills in the field	Pre-training, 3 months and 6 months post-training, but only a single post-training value is reported	BP; PSS; Impact of Events Scale total stress, intrusive and avoidance subscale scores; Response to Stressful Experience Scale; POQA subscales; on- and off-duty HR and HRV components (RMSSD, HF, LF, VLF, total power, LF/HF ratio), respiratory coherence on *n* = 26	Below threshold (*p* > 0.05) improvements to anger and resentment but increases in intrusive and avoidance scores. Significant post-training changes to sympathetic (LF) and parasympathetic (HF) contributors of HRV on both work and off days, increased RMSSD on off days, and significantly increased respiratory coherence. Coherence values not reported and requested from the authors
Ranta, 2009 [86] (Low)*	80	Police officers (India)	RCT	Multimodal psychophysiological stress management vs. Relaxation only	3 × 1 h sessions + brief home assignments vs. 1 × 1 h session	Pre-training, post-training	PSQ and CBQ global scores	Significant post-training improvements in both outcomes for the multimodal group only. SDs not provided, authors contacted.
Rø et al., 2010 [87] (Moderate-Low)	153	Nurses (Norway)	Prospective cohort study	Multimodal psychoeducational and psychophysiological retreat	5 days	Pre-training, 12 months post-training	MBI EE, DP, and PA subscales; proportion on sick leave; preceding year number of weeks on sick leave; adverse life events	Significant reductions in MBI EE and DP 12-months post-training. Number of adverse life events not reported, authors contacted
Rodrigues et al., 2018 [88] (Moderate-Low)	33	Nurses (USA)	Prospective cohort study	Stress management coping skill building	Single 90-min group session	Pre-training, 3 months post-training	MBI EE and DP subscales	Significant reduction in EE and DP 3 months post-training
Steinberg et al., 2016 [89] (Moderate-Low)*	32	SICU personnel (USA)	RCT	Mindfulness-based intervention	8 × 1 h weekly sessions + recommended 20-min practice × 5/week vs. Nil treatment	Pre-training, post-training	MBI EE, DP and PA subscales, ProQoL CS, BO, STS subscales, number of missed work days in past 2 months	Work satisfaction measures were significantly correlated with some mental health subscales, but were not reported or analyzed separately in the study. M and SDs for primary outcome measures not provided, authors contacted.
Tveito & Eriksen, 2009 [90] (Moderate-Low)	40	Nursing home employees (Norway)	RCT	Multimodal stress management coping skill building (IHP) vs. WLC	15 × 1 h weekly sessions and workplace assessment + 9 months physical exercise	Pre-training, post-training, 12 months	General Health Status Inventory SF-36 Mental Health subscale; Demand/Control Model subscales; number of days on sick leave; job stress (undefined)	No significant differences between groups post-training or 1-year follow-up
Villani et al., 2013 [91] (Moderate-Low)	30	Oncology nurses (Italy)	RCT	Web-based stress management coping skill building (M-SIT) vs. Neutral stimuli control group	15-min video clips, 2×/week, 4 weeks (8 sessions, 2 h total)	Pre-training, post-training	STAI; COPE Active coping and Denial subscales	Significant post-training improvement on all measures for M-SIT group only

*AAQ-II* Acceptance and Action Questionnaire II, *ACT-SMI* Acceptance and Commitment Therapy and Preventative Stress Management Intervention, *AUC* Area Under the Curve (Diurnal Cortisol), *AUDIT* Alcohol Use Disorders Identification Test, *BAI* Beck Anxiety Inventory, *BDI* Beck Depression Inventory, *BO* Burnout Subscale (ProQoL), *BP* blood pressure, *BRS* Brief Resilience Scale, *BSI* Brief Symptom Inventory, *CBQ* Coping Behaviour Questionnaire, *CDRS* Connor Davidson Resilience Scale, *CES-D* Center for Epidemiological Studies Depression Scale, *CF* Compassion Fatigue Subscale (ProQoL), *CFQ* Cognitive Fusion Questionnaire, *COPE* Brief Coping Orientation to Problems Experienced, *CS* Compassion Satisfaction Subscale (ProQoL), *DASS* Depression Anxiety Stress Scale-21, *DHEA* dehydroepiandrosterone-sulfate, *DP* Depersonalization Subscale (MBI), *EE* Emotional Exhaustion Subscale (MBI), *ERSQ* Emotion-Regulation Skills Questionnaire, *EZ* ERASE-Stress New Zealand, *FMI* Freiburg Mindfulness Inventory, *GHQ* General Health Questionnaire, *HADS* Hospital Anxiety and Depression Scale, *HF* high frequency, *HPLP-II* Health-Promoting Lifestyle Profile II, *HR* heart rate, HR_Avg_ average heart rate, *HR*_*Base*_ baseline resting heart rate, *HR*_*Index*_ maximum heart rate relative to resting heart rate, *HR*_*Max*_ maximum heart rate, *HR*_*Rec*_ recovery time from maximum to resting heart rate, *HRV* heart rate variabilitym, *HRV-BF* heart rate variability biofeedback, IBI interbeat intervals, *IHP* Integrated Health Program, *iTEC* Integrative Training of Emotion-Regulation Competencies, *LET* Life Engagement Test, *LF* low frequency, *LOT-R* Life Orientation Test-Revised, *M* mean, *MBI* Maslach Burnout Inventory, *ME* medical examiner, *METI* Managing Emergencies and Traumatic Incidents Organizational Program, *MHC-SF* Mental Health Continuum Short Form, *OLBI* Oldenburg Burnout Inventory, *PA* Personal Accomplishment Subscale (MBI), *PANAS* Positive Affect Negative Affect Scale, *Pbse* Performance-based Self-esteem Scale, *PCL* Posttraumatic Checklist, *PDS* Posttraumatic Diagnostic Scale, *POMS* Profile of Mood States, *POMS-TA* POMS Tension-Anxiety Subscale, *POQA* Personal and Organizational Quality Assessment, *PROMIS* Patient Reported Outcomes Measurement Information System, *ProQoL* Professional Quality of Life Scale, *PSQ* Police Stress Questionnaire, *PSS* Perceived Stress Scale, *RAW* Resilience@Work, *RCT* randomized control trial, *RMSSD* root mean squared standard deviation, *RS* Resilience Scale, *R2MR* Road to Mental Readiness, *SCL-90* Symptoms Checklist, *SCS* Self-Compassion Scale, SD Standard Deviation, *SICU* surgical intensive care unit, *SLS* Satisfaction with Life Scale, *STAI* State-Trait Anxiety Inventory, *STS* Secondary Traumatic Stress Subscale (ProQoL), *TAU* training as usual, VLF very low frequency, WLC waitlist control

### Study characteristics

The 42 studies represented data from 3182 individuals. Police officers were the most common PSP group (*n* = 15), followed by correctional workers (*n* = 2), firefighters (*n* = 2), and paramedical students (*n* = 1). There were no eligible studies, including participants from other PSP sectors. FHP occupations represented included nurses (*n* = 11) and various groupings of FHP (*n* = 9), including social workers, radiologists, medical examiners, physicians, nursing home employees, Red Crescent/Red Cross personnel, physiotherapists, occupational therapists, and healthcare clerical and administrative staff. The systematic literature search also yielded two relevant studies on educational staff exposed to a PPTE [60] and on miners whose occupational roles pose a realistic and substantial life threat (e.g., workers, blasters, foremen at the mine face) [82].

PTSI mitigation program themes identified in the eligible studies overlapped heavily and are not mutually exclusive (i.e., one program may fall under multiple themes). The aggregation of programs into broader categories was conducted to perform the meta-analysis and are defined in Table 2. Program themes included mindfulness (*n* = 13), psychoeducation (*n* = 20), psychophysiology (*n* = 11), resilience promotion (*n* = 17), stress management (*n* = 15), building coping skills (*n* = 7), emotion regulation (*n* = 4), cognitive (behavior) therapy (*n* = 3), and psychosocial support or counseling (*n* = 2). There were 8 studies that evaluated the effectiveness of self-described “multimodal programs” that included multiple themes identified above. There were 6 studies that employed biofeedback with primary resilience promoting program types.

Study designs included RCTs (including cluster, parallel, and crossover RCTs) (*n* = 26) or prospective cohort studies (*n* = 15). Comparators included waitlist controls (i.e., offered the program at the end of the study) (*n* = 9), no training or occupational skills training (i.e., not mental health training) as usual (*n* = 11), psychoeducation only (*n* = 2), neutral or negatively valenced versions of the program (*n* = 2), or alternative control programs such as general wellness (*n* = 1) or Critical Incident Stress Management (*n* = 1). There were 7 studies that used online or web-based presentation of their programs, while 34 studies used in-person group sessions. Program durations ranged from a single 90 minute group session [88] or one-day workshop or equivalent (i.e., less than 8 hours) (*n* = 6) to 4- or 5-day workshops or retreats [35, 36, 40, 87]. Multiple training sessions were distributed over a minimum of two days [79] and a maximum of 9 months [90], which included 15 weeks of mental health programming within nine months of recommended physical exercise. Self-paced programs (*n* = 8) were predominantly web-based, and studies reported very low levels of program completion and/or adherence [62, 64, 74]. Several programs, predominantly mindfulness-based, also included optional or recommended daily practice, or “homework” (*n* = 19). Study duration for follow-up evaluations ranged from immediately following the training program (*n* = 25), 1 week to 3 months post-training (*n* = 16), 6 months (*n* = 6), 7 to 12 months (*n* = 8), and 13 to 18 months (*n* = 3). A single study with multiple follow-up durations would be included in more than one of the categories (e.g., Andersen et al. [40] conducted pre-, post-training, 6-, 12-, and 18-month evaluations).

Due to the wide variety and limited consistency in PTSI mitigation program types, outcome variables, occupational groups, and follow-up durations across studies, the meta-analysis results will be presented by outcome categories (defined in Table 3) and are summarized in Table 5. Below, we report on the effectiveness of PTSI mitigation programs on reducing symptoms of PTSI and improving general measures of psychological health and wellness. Effect sizes (*SMD*) and confidence intervals (CIs) will be reported for specific outcomes. Any statistically significant differences in outcomes by program type, follow-up duration, and/or occupational group will then be presented where subgroup analyses were performed. Supporting figures can be found in **Additional File** 2.

**Table 5:** Summary of meta-analytic results, subgroup analyses, and publication biases

Outcome	Studies	***Meta-analytic results***	***Subgroup analyses***				***Publication biases***
		***SMD*** [95% CI]	Timepoint	Program type	Occupation	Study design	Linear regression test of funnel plot asymmetry
Absenteeism	4	0.01 [− 0.19; 0.21]	–	–	–	–	–
Alcohol	10	− 0.08 [− 0.21; 0.06]	–	–	–	–	–
Antithrombin	1	0.49 [− 0.45; 1.44]	–	–	–	–	–
Anxiety	21	− 0.20 [− 0.31; − 0.10]	0.61	0.31	0.76	–	− 0.10 [− 0.23; 0.02], *p* < 0.01 *
Blood pressure	8	− 0.17 [− 0.38; 0.04]	–	–	–	–	–
Burnout	15	− 0.45 [− 0.64; − 0.26] *	0.15	0.04*	0.46	–	− 0.28 [− 0.47; − 0.09], *p* = 0.07
Coping	5	0.41 [0.02; 0.80] *	< 0.01*	< 0.01*	< 0.01*	–	–
Cortisol	6	− 0.20 [− 0.44; 0.04]	–	–	–	–	–
Depression	18	− 0.46 [− 0.71; − 0.21] *	0.05*	< 0.01*	0.80	0.88	− 0.17 [− 0.47; 0.12], *p* = 0.03 *
DHEA	2	− 0.29 [− 0.80; 0.21]	–	–	–	–	–
Drug use	2	− 0.05 [− 0.35; 0.24]	–	–	–	–	–
General symptoms	7	− 0.70 [− 1.14; − 0.26] *	< 0.01*	< 0.01*	0.46	–	–
Heart rate	21	− 0.27 [− 0.40; − 0.14]	0.04*	0.01*	0.04*	–	− 0.23 [− 0.37; − 0.09], *p* = 0.10
Prolactin	1	− 0.07 [− 0.56; 0.41]	–	–	–	–	–
PTSD	9	− 0.33 [− 0.55; − 0.11] *	< 0.01*	0.10	0.03*	–	–
Resilience	22	0.27 [0.13; 0.42] *	0.02*	< 0.01*	0.29	0.78	0.27 [0.13; 0.42], *p* = 0.62
Stress	25	− 0.35 [− 0.51; − 0.20] *	0.49	0.14	0.02*	0.80	− 0.21 [− 0.37; − 0.05], *p* = 0.55
Suicidality	2	0.33 [− 0.07; 0.73]	–	–	–	–	–
Well-being	20	0.46 [0.26; 0.66] *	0.63	0.24	< 0.01*	0.45	0.46 [0.26; 0.66], *p* = 0.16

The standardized mean difference (*SMD*) is a method of pooling continuous outcomes (i.e., scores on rating scales) in meta-analysis. It is preferred over mean differences when there are differences in how the outcome is measured across studies. The asterisk (*) indicates that the pooled estimate is statistically significant at *p* < 0.05 (i.e., the confidence interval (CI) does not overlap with the null). Subgroup analyses were only performed where at least ten studies were included. Statistically significant subgroup analyses (*p* < 0.05) are marked with an asterisk (*). The linear regression test of funnel plot asymmetry is used to appraise publication bias in the pooled estimate for any individual outcome in the meta-analysis. *p* values smaller than 0.05 (marked with an asterisk) are considered statistically significant and indicate evidence of publication bias in that outcome’s pooled estimate. *DHEA*, dehydroepiandrosterone; *PTSD*, posttraumatic stress disorder

### Mental disorder symptoms and absenteeism

#### Depression

Significant reductions in mental disorder symptoms were observed for depression, with an *SMD* of − 0.46 [ − 0.71; − 0.21]. Depression effect sizes were largest for resilience promotion programs (*SMD* = − 1.05; *p* < 0.01) and immediately post-training (− 0.78) compared with follow-up (*p* = 0.05) (**Figure 2.1 in Additional File** 2).

#### Burnout

Moderate reductions in symptom burden were also observed for burnout (*SMD* = − 0.45 [− 0.64; − 0.26]), with larger effect sizes observed in resilience promotion versus multimodal programs (− 0.90 vs. − 0.24; *p* = 0.04) (**Figure 2.2 in Additional File** 2).

#### PTSD

Medium reductions were observed for PTSD symptoms with an *SMD* of − 0.33 [− 0.55; − 0.11]. Significant subgroup analyses evidenced the largest effect sizes at 8-month follow-up (− 1.22; *p* < 0.01), and among PPTE-exposed educators (− 0.86; *p* = 0.03) (**Figure 2.3 in Additional File** 2).

#### Anxiety

Small reductions were observed across all studies for anxiety symptoms, with an *SMD* of − 0.20 [− 0.31; − 0.10].

#### Suicidality

Suicidality was not significantly reduced with the programs considered (*SMD* = 0.33 [− 0.07; 0.73]).

#### Substance use

For alcohol, the overall reduction in weekly alcoholic drinks was small and not statistically significant, with an *SMD* of − 0.08 [− 0.21; 0.06]. The effect was even smaller for other substance use, with an *SMD* of − 0.05 [− 0.35; 0.24]. Given the small study yield for either outcome (Table 5), subgroup analyses were not conducted.

#### Absenteeism

There was no significant improvement in absenteeism—defined as the number of sick days taken by employees in the study—with an *SMD* of 0.01 [− 0.19; 0.21].

### General measures of general psychological health, stress, resilience, and well-being

#### General psychological health

There was a significant medium reduction in general psychological symptom burden across studies, with an *SMD* of − 0.70 [− 1.14; − 0.26] (**Figure 2.4 in Additional File** 2). There were significant differences in effect sizes across timepoints (*p* < 0.01) – with small positive effects (i.e., increases in general psychological symptoms relative to pre-training) noted at 18-month follow-up (0.34) relative to large decreases in symptoms (reflected by negative effect sizes) at 1-month (− 0.95) and immediate post-training (− 0.91) timepoints—and by program type (*p* < 0.01), with the larger reductions associated with multimodal programs (− 1.09) relative to resilience promotion programs (0.34).

#### Stress

Stress symptoms were associated with small-to-medium reductions in symptom burden overall, with an *SMD* of − 0.35 [− 0.51; − 0.20] and larger effects among hospital staff (− 0.84) compared with police officers (− 0.29; *p* = 0.02).

#### Well-being

Among measures of broader mental health status, effect sizes were largest for well-being with a medium *SMD* of 0.46 [0.26; 0.66]; larger effects were observed among educators (1.95) compared with other occupational categories (*p* < 0.01).

#### Coping

There was evidence of a medium *SMD* of 0.41 [0.02; 0.80], with larger effect sizes at 18-months (0.93) versus other timepoints (*p* < 0.01), with resilience promotion programs (0.93) relative to other program themes (*p* < 0.01), and among police officers (0.73) and radiation therapists and nurses (0.70) relative to paramedical staff (− 0.01; *p* < 0.01) (**Figure 2.5 in Additional File** 2).

***Resilience:*** Overall improvement in resilience was small, with an *SMD* of 0.27 [0.13; 0.42] (**Figure 2.6 in Additional File** 2). Effect sizes were largest at immediate post-test (− 0.46; *p* = 0.02) and larger with resilience-promoting strategies (0.98) relative to other modalities (*p* < 0.01).

### Biological measures of stress

There was no evidence that the investigated programs reduced serum biomarkers of stress, such as cortisol, antithrombin, dehydroepiandrosterone (DHEA), and prolactin (Table 5). While there were no significant reductions in blood pressure across studies, there were significant improvements in overall and average measures of heart rate, with a small *SMD* of − 0.27 [− 0.40; − 0.14]. The greatest reductions in heart rate were seen at 12-months of follow-up (− 1.52, *p* = 0.04), with resilience promotion programs (− 1.00; *p* < 0.01), and among nurses (− 0.55) and police officers (− 0.45) compared with correctional officers (− 0.15; *p* = 0.04).

### Publication bias

There was evidence of a publication bias for anxiety (*p* = 0.0061) and depression (*p* = 0.03) (**Figure 2.7 in Additional File** 2). The trim-and-fill method was used to account for potential outcome effect size estimate inflation (Table 5). For anxiety, the effect size changed from an *SMD* of − 0.20 to − 0.10 [− 0.23; 0.02]. For depression, the effect size changed from an *SMD* of − 0.46 to − 0.17 [− 0.47; 0.12]. For each outcome, correction for publication bias significantly reduced the effect size, meaning that anxiety and depression outcomes are likely associated with publication bias.

### Quality assessment

**Fig. 2:**
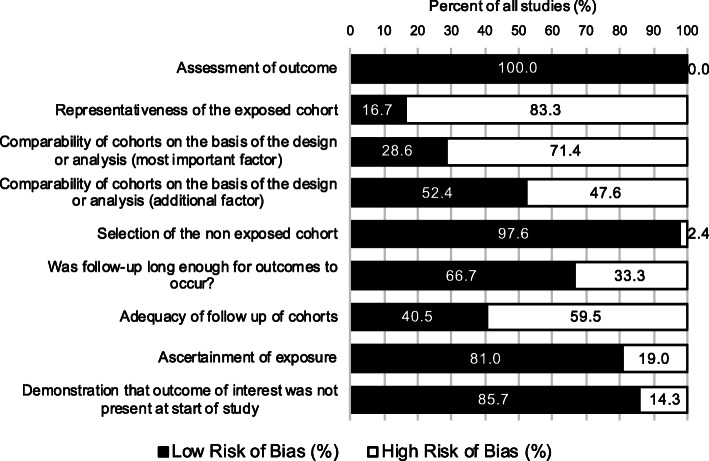
Quality assessment using Newcastle-Ottawa Scale. Full sample (*n* = 36 studies) summary of strength of evidence from systematic review and meta-analysis

Quality assessment ratings for all studies in the current systematic review are illustrated in Fig. 2. Individual study ratings are reported in Table 4, and detailed study ratings are presented in **Table 3.1** in **Additional File** 3. Overall, only one study was of “high quality” [63], 11 were “moderate to high quality,” 24 were of “moderate to low quality,” and six were of “low quality.”

#### Outcome

All studies were rated at low risk of bias because all used empirically validated self-report mental disorder screening tools or objective physiological data. There were 14 studies that were rated at high risk of bias for follow-up periods; outcomes were only assessed immediately after program delivery, prohibiting evaluations of program effectiveness following subsequent occupational exposures to PPTE. Concerning adequacy of follow-up, more than half of all studies (*n* = 25) received high risk of bias ratings for either failing to report post-training sample sizes and any possible participant attrition and/or failing to provide analyses of retained participants to those lost at follow-up.

#### Selection

All but seven studies were rated at a high risk of selection bias for failing to demonstrate sample representativeness, limiting results’ generalizability. All studies were rated at a low risk of bias due to clear selection criteria for control groups except for one sample from a different police district [85]. Most studies adequately ascertained participation in the programming being assessed (*n* = 34); however, three studies were either unclear about program completion or participation [68, 77, 86] and five studies of self-paced online programs reported very low participation or completion [62, 64, 74–76].

#### Comparability

The purpose of the current systematic review was to identify the effectiveness of proactive psychological programs designed to mitigate PTSI and limit the decline of psychological symptoms among workers at high risk of exposure to a PPTE. Therefore, studies that included individuals with diagnosed PTSI or mental health disorders were excluded at the title and abstract screening phase, as these would be considered PTSI treatments or services. Accordingly, the comparability criterion pertains to controlling for the most important factor in the study design, which in the case of the current review is the presence of a pre-existing PTSI (i.e., before program onset) and/or exposure to a PPTE following program onset, both of which would significantly confound investigations of program effectiveness. Only 12 of the included studies received a low risk of bias rating for the comparability criterion, either reporting or controlling for mental disorder symptom severity at baseline or pre-training measures, or by reporting PPTE exposures following program onset and before any follow-up evaluations. All remaining studies (*n* = 25) were at a high risk of bias.

Conversely, all but five studies received a low-risk rating for demonstrating that the outcome of interest was not present at the start of the study by providing pre-training baseline measures for reported outcomes. Roughly half of the eligible studies (*n* = 22) controlled an additional factor in the study design or analysis, including age or years of service/employment, which are known correlates of mental health among PSP and FHP [4, 14, 92].

## Discussion

The effectiveness of various organizational programs designed to “prevent”—or more accurately to proactively mitigate—PTSI and improve psychological health indicators among PPTE-exposed occupational groups remains unclear. The current systematic review identified 42 empirical research studies measuring the effectiveness of organizational training programs designed to proactively mitigate PTSI among PSP, FHP, and other workers exposed to PPTEs (Table 4). A great deal of heterogeneity was indicated across program themes and durations and study designs, durations, and follow-up periods. Self-directed or web-based programs also suffered from poor participant adherence and completion. The quality assessment indicated a high risk of reporting bias for several study elements (Fig. 2), including failure to demonstrate sample representativeness (83% of studies), evaluate or report on the presence of a PTSI and/or mental disorder before the study and program onset, and/or participant exposure to PPTEs before follow-up evaluations (71%). The identified factors would significantly confound investigations of program effectiveness and limit the generalizability of results. Most studies also reported high attrition rates at follow-up evaluations (60%), and several collected post-training measures before the newly acquired skills could be practiced or applied in work conditions (33%).

Evaluation of 36 study outcomes with a quantitative meta-analysis provide evidence that all programs (i.e., collapsed across program type) resulted in statistically significant reductions in PTSI after training (Table 5), including symptoms of general psychological health, depression, burnout, stress, PTSD, and anxiety, as well as significant improvements in measures of well-being, coping, and resilience (see Tables 2 and 3 for operational definitions of program types and outcomes, respectively). Consistent with previous literature [13, 41, 42], post-training improvements are of a medium (*SMD* < 0.8) or small (*SMD* < 0.5) effects in magnitude and time-limited. Subgroup analyses indicated large (*SMD* > 0.8) effect sizes for sustained improvements in PTSD symptoms and coping for up to 18 months. Resilience promotion programs appeared to reduce symptoms of depression and burnout and improved coping and resilience measures. Multimodal programs that combined therapeutic approaches (e.g., mindfulness, stress management, emotion regulation, resilience promotion) appeared to improve measures of general psychological health. Police appeared to report the greatest improvement in coping measures, likely due to the overrepresentation of this population across studies (*n* = 15). In contrast, combined groups of FHP indicated the greatest reduction in stress symptoms, and PPTE-exposed educational staff indicated the greatest decrease in PTSI symptoms [61]. Significant and sustained improvements in coping may be promising, but coping is not yet a clinically validated construct for PTSI. Similarly, wellness and resilience are general health indicators that vary in operational and theoretical definitions between individual studies. Substantial barriers to evaluating program effectiveness identified in the current review include inconsistency in outcome measures, outcome reporting (i.e., mean and standard deviation values required for meta-analysis), and follow-up durations across studies. The limitations precluded more detailed subgroup analyses and data synthesis for the current meta-analysis.

Despite claims for reduced absenteeism as a justification for funding and implementation of mental disorder “prevention” programs, evidence provided by the current meta-analysis does not support reduced absenteeism due to inconsistent or insufficient reporting, and especially for distinguishing missed work as a result of a psychological injury sustained at work or due to physical illness. Similarly, the results did not evidence statistically significant reductions in substance use or suicidality, both of which are prevalent among PSP [4, 14, 21] but remain under-investigated among FHP and other at-risk workers. There was also no evidence for post-training improvements in physiological biomarkers of stress except for heart rate (Table 5), which was the third most common outcome measure and included several studies that condition adaptive through heart rate variability biofeedback training (HRV-BF). Despite the limited evidence supporting the effectiveness of HRV-BF as a possible tool for modulating stress physiology, doing so requires substantial resources such as medical-grade cardiovascular equipment, software, repeated training sessions, and qualified personnel psychophysiology to guide psychoeducational modules [35, 36, 40, 79, 80].

The studies identified by the current systematic literature review provide modest evidence for very time-limited improvements in several PTSI symptoms. Variability in program types and durations also represents variability in organizational cost and feasibility, which place limitations on implementing and repeated investigation of PTSI mitigation program effectiveness either separate from or part of a longitudinal research study (see also [93]). Web-based or self-directed programs may be considered more cost-effective to implement than multiple in-person group sessions; however, the current systematic review results demonstrate a critical and substantial lack of adherence, as well as very low completion rates for several online program protocols [62, 64, 74–76]. Inconsistent and poor-quality study designs precluded more conclusive recommendations directed at organizational stakeholders to inform PTSI mitigation programming or training tailored to PSP and FHP.

## Limitations

Despite a relatively high number of eligible studies (*n* = 42), the main limitation of the current systematic review is the high heterogeneity across studies, which precluded the inclusion of six studies in a quantitative meta-analysis and more detailed subgroup analyses. The quality of available studies was also highly variable (Fig. 2 and **Table 3.1 in Additional File** 3), with 71% (30 of 42) of studies scoring moderate to low quality (≤ 6 out of 9). The geographical variability of participants also makes generalizability difficult because an effective program in one political, cultural, social, economic, and epidemiological context may not be relevant, applicable, or effective elsewhere. Nevertheless, the substantial impact of PPTE exposures on the mental health of PSP and FHP appears broadly accepted, as does the need to develop effective evidence-based PTSI mitigation programming for all at-risk workers to minimize personal, social, and economic costs [26, 29].

Publication bias was high for studies with outcome measures assessing anxiety and depression, and adjustments for methods biases rendered the results no longer statistically significant (Table 5). As smaller trials are generally analyzed with less methodological rigor than larger ones, the resulting asymmetrical funnel plot suggested that selective reporting may have led to an overestimation of effect sizes in smaller trials (**Figure 2.7 in Additional File** 2). Other limitations included the search strategy and criteria process (Table 1), which was restricted to English- and French-language studies published after 2008 from five indexed electronic databases.

## Conclusions

Especially during the current global coronavirus pandemic, there is an urgent need to identify effective organizational training tailored for PSP and FHP and designed to mitigate the psychological impact of PTSI that can result from occupational PPTE exposures [26, 29]. The extant literature identified by the current systematic literature review indicates broad variety in sampled occupational populations, implemented programming approaches, and measured outcome variables. Heterogeneity across studies precludes identifying a proactive PTSI mitigation program type that is superior to others and effective for diverse PSP, FHP, and other at-risk workers exposed to PPTE. Nonetheless, we have synthesized the available evidence on proactive programming effectiveness in reducing specific PTSI symptoms. Based on our meta-analytic results, resilience promotion and multimodal programs that combine a variety of therapeutic and skill-building approaches appear to produce modest time-limited reductions in symptoms of general psychological health, depression, burnout, stress, PTSD, and anxiety, as well as promoting well-being, adaptive coping, and resilience. By identifying significant research gaps and practical study limitations, we intend to help inform future high-quality research evaluating program effectiveness within the context of PSP and FHP working environments. The current results provide organizational stakeholders and policymakers with numerous options for developing innovative mental health solutions tailored to the unique occupational challenges faced by those who serve to maintain and protect public health and safety.

## Supplementary Information

**Additional file 1.**

**Additional file 2.**

**Additional file 3.**

## Data Availability

The datasets used and/or analyzed during the current study are available from the corresponding author on reasonable request.

## Abbreviations

DHEA: Dehydroepiandrosterone
FHP: Frontline healthcare professional
HRV-BF: Heart rate variability biofeedback
PPTE: Potentially psychologically traumatic event
PSP: Public safety personnel
PTSD: Posttraumatic stress disorder
PTSI: Posttraumatic stress injury
SMD: Standardized mean difference
