# Supplementary material for: Proactive psychological programs designed to mitigate posttraumatic stress injuries among at-risk workers: a systematic review and meta-analysis
Source: Syst Rev. 2021 Apr 28;10:126. doi: 10.1186/s13643-021-01677-7 (PMC8079856; doi:10.1186/s13643-021-01677-7)
Supplement: Supplementary file 2 — Additional file 2. [file 13643_2021_1677_MOESM2_ESM.docx]

*Figure 2.1.* Forest plots for depression symptoms. A. Outcome analysis for depression across studies. B. Subgroup analyses for depression by program type (*p* < 0.01). C. Subgroup analyses for depression by timepoint (*p* = 0.05).

***Figure 2.2***. Forest plots for burnout symptoms. A. Outcome analysis for burnout across studies. B. Subgroup analyses for burnout by program type (*p* = 0.04).

***Figure 2.3***. Forest plots for PTSD symptoms. A. Outcome analysis for PTSD across studies. B. Subgroup analyses for PTSD by timepoint (*p* < 0.01).

***Figure 2.4***. Forest plots for general psychological health symptoms. A. Outcome analysis for general psychological health across studies. B. Subgroup analyses for general psychological health by timepoint (*p* < 0.01). C. Subgroup analyses for general psychological health by program type (*p* < 0.01).

***Figure 2.5***. Forest plots for coping. A. Outcome analysis for coping across studies. B. Subgroup analyses for coping by timepoint (*p* < 0.01). C. Subgroup analyses for coping by program type (*p* < 0.01). D. Subgroup analyses for coping by occupational group (*p* < 0.01).

***Figure 2.6***. Forest plots for resilience. A. Outcome analysis for resilience across studies. B. Subgroup analyses for resilience by program type (*p* < 0.01).

***Figure 2.7.*** Funnel plots for publication bias. A. Funnel plot for anxiety. B. Funnel plot for depression.
