# Supplementary material for: Proactive psychological programs designed to mitigate posttraumatic stress injuries among at-risk workers: a systematic review and meta-analysis
Source: Syst Rev. 2021 Apr 28;10:126. doi: 10.1186/s13643-021-01677-7 (PMC8079856; doi:10.1186/s13643-021-01677-7)
Supplement: Supplementary file 3 — Additional file 3. [file 13643_2021_1677_MOESM3_ESM.docx]

Table 3.1. Quality assessment using Newcastle-Ottawa scale– individual study assessments (n=42).

| **Study** | **Assessment of outcome** | **Representativeness of the exposed cohort** | **Comparability of cohorts (PTSI)** | **Comparability of cohorts (other factor)** | **Selection of non- exposed** | **Duration of follow-up** | **Adequacy of follow-up** | **Ascertainment of exposure** | **Primary outcome not present at baseline** |
| --- | --- | --- | --- | --- | --- | --- | --- | --- | --- |
| Alexander 2015 | low | high | high | low | low | high | high | low | low |
| Andersen 2015 | low | low | high | high | low | high | high | low | low |
| Andersen 2016 | low | low | high | low | low | high | low | low | low |
| Andersen 2018 | low | high | high | high | low | low | low | low | low |
| Anderson 2017 | low | high | high | low | low | low | high | low | low |
| Arble 2017 | low | high | high | low | low | low | low | low | low |
| Arnetz 2009 | low | high | high | high | low | low | high | low | unclear |
| Arnetz 2013 | low | low | high | low | low | low | low | low | low |
| Bademci 2016 | low | high | high | low | low | high | high | low | low |
| Berger 2016 | low | high | low | low | low | low | high | low | low |
| Berking 2010 | low | high | high | high | low | high | low | low | low |
| Bolier 2014 | low | high | low | high | low | low | high | high | low |
| Brinkborg 2011 | low | low | low | low | low | low | low | low | low |
| Brondolo 2017 | low | high | high | low | low | low | high | high | low |
| Carleton 2018 | low | high | high | high | low | low | high | low | low |
| Cheng 2015 | low | high | low | low | low | low | low | low | high |
| Chongruksa 2012 | low | high | low | low | low | low | high | low | low |
| Christopher 2016 | low | high | high | high | low | high | high | low | low |
| Christopher 2018 | low | high | high | low | low | low | low | unclear | low |
| Craigie 2016 | low | high | low | high | low | low | low | low | low |
| Daigle 2018 | low | high | low | high | low | high | high | low | low |
| Duarte 2017 | low | high | high | high | low | high | high | low | high |
| Duchemin 2015 | low | high | high | low | low | low | low | low | high |
| Flarity 2013 | low | high | low | high | low | high | high | low | low |
| Hersch 2016 | low | high | high | low | low | low | low | high | low |
| Joyce 2018 | low | high | high | high | low | high | high | high | low |
| Joyce 2019 | low | high | low | low | low | low | low | high | low |
| Larijani 2018 | low | high | high | high | low | high | high | high | high |
| Lin 2019 | low | high | high | high | low | low | high | low | low |
| McCraty 2009 | low | high | low | low | low | low | high | low | low |
| McCraty 2012 | low | high | high | low | low | low | high | low | low |
| Mealer 2014 | low | high | low | high | low | high | low | low | low |
| Molek-Winiarska 2018 | low | low | high | high | low | low | low | low | low |
| Oliver 2009 | low | low | high | low | low | low | low | low | low |
| Poulsen 2015 | low | high | high | low | low | low | high | low | low |
| Ramey 2016 | low | high | high | low | unclear | low | high | low | low |
| Ranta 2009 | low | high | high | high | low | high | high | high | low |
| Ro 2010 | low | high | high | low | low | low | high | low | low |
| Rodrigues 2018 | low | high | high | high | low | low | low | low | low |
| Steinberg 2016 | low | low | high | low | low | low | high | low | high |
| Tveito 2009 | low | high | high | high | low | low | low | low | low |
| Villani 2013 | low | high | low | high | low | high | high | low | low |
